# Regional variation and temporal trends in transcatheter and surgical aortic valve replacement in Switzerland: A population-based small area analysis

**DOI:** 10.1371/journal.pone.0296055

**Published:** 2024-01-08

**Authors:** Carla Schenker, Maria M. Wertli, Lorenz Räber, Alan G. Haynes, Arnaud Chiolero, Nicolas Rodondi, Radoslaw Panczak, Drahomir Aujesky

**Affiliations:** 1 Department of General Internal Medicine, Inselspital, Bern University Hospital, University of Bern, Bern, Switzerland; 2 Department of Internal Medicine, Cantonal Hospital Baden, Baden, Switzerland; 3 Department of Cardiology, Inselspital, Bern Bern University Hospital, University of Bern, Bern, Switzerland; 4 CTU Bern, University of Bern, Bern, Switzerland; 5 Institute of Primary Health Care (BIHAM), University of Bern, Bern, Switzerland; 6 School of Population and Global Health, McGill University, Montreal, Canada; 7 Population Health Laboratory (#PopHealthLab), University of Fribourg, Fribourg, Switzerland; 8 Institute of Social and Preventive Medicine, University of Bern, Bern, Switzerland; Nanjing Drum Tower Hospital: Nanjing University Medical School Affiliated Nanjing Drum Tower Hospital, CHINA

## Abstract

**Background:**

Aortic valve stenosis (AS) is the most common valvular heart disease and if severe, is treated with either transcatheter (TAVR) or surgical aortic valve replacement (SAVR). We assessed temporal trends and regional variation of these interventions in Switzerland and examined potential determinants of geographic variation.

**Methods:**

We conducted a population-based analysis using patient discharge data from all Swiss public and private acute care hospitals from 2013 to 2018. We generated hospital service areas (HSAs) based on patient flows for TAVR. We calculated age-standardized mean procedure rates and variation indices (extremal quotient [EQ] and systematic component of variation [SCV]). Using multilevel regression, we calculated the influence of calendar year and regional demographics, socioeconomic factors (language, insurance status), burden of disease, and number of cardiologists/cardiovascular surgeons on geographic variation.

**Results:**

Overall, 8074 TAVR and 11,825 SAVR procedures were performed in 8 HSAs from 2013 to 2018. Whereas the age-/sex-standardized rate of TAVR increased from 12 to 22 procedures/100,000 persons, the SAVR rate decreased from 33 to 24 procedures during this period. After full adjustment, the predicted TAVR and SAVR rates varied from 12 to 22 and 20 to 35 per 100,000 persons across HSAs, respectively. The regional procedure variation was low to moderate over time, with a low overall variation in TAVR (EQ 1.9, SCV 3.9) and SAVR (EQ 1.6, SCV 2.2). In multilevel regression, TAVR rates increased annually by 10% and SAVR rates decreased by 5%. Determinants of higher TAVR rates were older age, male sex, living in a German speaking area, and higher burden of disease. A higher proportion of (semi)private insurance was also associated with higher TAVR and lower SAVR rates. After full adjustment, 10.6% of the variance in TAVR and 18.4% of the variance in SAVR remained unexplained. Most variance in TAVR and SAVR rates was explained by language region and insurance status.

**Conclusion:**

The geographic variation in TAVR and SAVR rates was low to moderate across Swiss regions and largely explained by differences in regional demographics and socioeconomic factors. The use of TAVR increased at the expense of SAVR over time.

## Background

Aortic valve stenosis (AS) is the most common valvular heart disease and its prevalence increases with age [1]. Overall, 12.4% of persons aged ≥75 years have AS, of whom about one fourth have severe AS [1]. Approximately 50% of patients with severe AS develop symptoms (e.g., dyspnea, angina, dizziness) and 53% undergo valve replacement within five years [2]. In both symptomatic and severe asymptomatic AS, observational and randomized-controlled studies have shown a mortality reduction in patients who underwent surgical aortic valve replacement (SAVR) compared to conservative treatment [2,3]. Hence, guidelines recommend SAVR for severe symptomatic AS, severe asymptomatic AS with reduced left ventricular function, or moderate AS if cardiac surgery is needed for other indications [4,5].

Despite its benefits, the surgical risk of SAVR is deemed unacceptably high in about one third of patients with severe symptomatic AS [6,7]. Clinical trials published between 2012 and 2019 demonstrated that less invasive transcatheter aortic valve replacement (TAVR) appears to be at least as effective and safe as SAVR [8–18]. Further, patients with TAVR may have a shorter length of hospital stay and fewer readmissions [19,20]. While guidelines published in 2012/14 recommended TAVR for patients with a prohibitive surgical risk and a life-expectancy of >1 year [21,22], more recent guidelines recommend an individualized approach considering age, femoral access, comorbidities, frailty, mortality, and life expectancy [4,5,23].

Since the first approval of TAVR in 2007, the uptake of TAVR into clinical practice has increased rapidly across the world [24–26]. In the USA, the use of TAVR in elderly patients with AS exceeded SAVR for the first time in 2017 [26]. However, evidence suggest a large variation in the use of TAVR across and within nations [25–27]. These differences were attributed to differences in the demographics of patients, access to new technologies, resources, reimbursement, and local practice patterns [25,26,28]. According to registry and market tracking data, Switzerland had the second highest TAVR penetration rate (34.5%), defined as the actual TAVR use relative to potential use, among European countries between 2007 and 2011 [25]. Moreover, TAVR is covered by Swiss health insurers since 2013 [29]. Switzerland, with its relatively homogenous population and universal health care coverage, offers a unique opportunity to study the uptake and predictors of TAVR use. We thus assessed temporal trends and regional variation in TAVR and SAVR rates from 2013 to 2018 using population-based data from all Swiss acute care hospitals. We explored whether regional demographics, socioeconomic factors, burden of disease, and number of cardiologists/cardiovascular surgeons were associated with procedure use.

## Methods

### Data sources

We conducted a population-based, small area analysis using routinely collected patient discharge data from all Swiss public and private acute care hospitals and census data was conducted for calendar years 2013–2018. The methods used for this analysis have been described previously [30–33]. Swiss hospitals are legally obligated to provide the Swiss Federal Statistical Office (SFSO) with an anonymized, standardized data set for each hospital discharge. These data are then combined and centrally stored in the Swiss Hospital Discharge Masterfile. Recorded variables include patient age, sex, nationality, insurance status, the type of admission (emergency vs. elective), up to 100 procedure codes based on the Swiss Classification of Operations (CHOP, an adaptation of the U.S. ICD-9-CM volume 3 procedure classification) [34], and up to 50 diagnostic codes based on the International Classification of Diseases, 10^th^ revision, German Modification (ICD-10-GM). Further, the area of patient residence and hospital location within one of 705 Swiss MedStat regions are recorded. MedStat regions represent Swiss statistical regions based on aggregated ZIP-codes [35]. The SFSO reviews data integrity and completeness by means of a specifically designed software [36]. As hospital reimbursement is directly based on the documentation of the main procedures, data completeness and accuracy of CHOP codes is likely to be very high [37].

We used Swiss National Cohort data from 2014 to determine the main language (Swiss German, French, or Italian) [38] and data from the SFSO to determine the population density for each MedStat region. We used the average Swiss Socioeconomic Position (SSEP, version 2) as a measure of socioeconomic status. The SSEP version 2 was derived using 2012–2015 Swiss structural surveys data to rank Swiss neighborhoods based on four domains (median rent/m^2^, proportion of households with a person with no/low education, proportion of households with a person in manual/unskilled occupation, and mean crowding, all on the neighborhood level) [39]. The SSEP varies between zero (lowest) and 100 (highest) and correlates well with mortality [39]. We obtained the number of cardiologists and cardiovascular surgeons per MedStat region for calendar year 2014 from the Swiss Medical Association. Our study was based on anonymized administrative data only and was thus, exempted from ethics committee approval according to the Swiss Human Research Act.

### Derivation of TAVR and SAVR specific hospital service areas (HSA)

Switzerland has compulsory basic health insurance coverage, with voluntary semiprivate and private insurance plans covering additional medical services. Although Swiss hospital care is primarily organized based on 26 geographic regions (the cantons) patients may utilize hospital services outside their canton of residence and the use of cantons as a unit of observation may skew procedure rates. We therefore used a fully automated method to generate reproducible general hospital service areas (HSAs) using all patient discharge data from the calendar years 2013–2016 (data that was available when the general HSAs were derived) [31]. Briefly, we identified 4,105,885 discharges of patients aged ≥18 years living in Switzerland from 155 Swiss acute care hospitals during calendar years 2013–2016. Across the 705 Swiss administrative (MedStat) regions, we identified regions that contain one or several hospitals as potential HSAs. Starting from these potential HSAs, in a centrifugal stepwise approach, we identified the geographically neighboring MedStat regions and merged them with the HSA if the majority of its residents were discharged from hospitals located in the specific HSA (plurality rule) [40]. HSAs with <50% of the patients being treated within the same HSA or <10 discharges overall were merged with the neighboring HSA which received most discharges until ≥ 50% and ≥10 discharges occurred within each HSA. This process yielded 63 general HSAs.

We then identified patient discharges with specific CHOP codes for SAVR (35.21.00/10-12, 35.21.20/99, 35.22.00/20/21/30/99, 35.F1.09/11/12, 35.F1.21/22, 35.F1.31/32, 35.F1.41/42, 35.F1.51/52, 35.F1.61/62, 35.F1.71/72) and for TAVR (35.22.10–13/19, 35.96.11, 35.F1.23–25, 35.F1.33–35, 35.F1.43–45) from all Swiss acute care hospitals during calendar years 2013–2018. As TAVR and SAVR are performed in tertiary care hospitals only, we specifically analyzed patient flows for TAVR procedures. Using the procedure described above, HSAs with <50% of the procedures performed within this HSA or with <10 discharges for TAVR were further aggregated into 8 TAVR-specific HSAs. We then drew choropleth maps of the eight final HSAs using Geographical Information System (GIS)-compatible vector files.

### Study population

Overall, we identified 19,899 patient discharges who underwent at least one TAVR (N = 8074) and/or SAVR (N = 11,825) from 2013 to 2018. Discharges with both interventions (N = 32; i.e., TAVR and SAVR within the same hospital stay) were included in the TAVR group analysis only.

### Measures of variation

We calculated unadjusted and age- and sex-standardized TAVR and SAVR rates per 100,000 persons for each HSA using procedure counts and 2013–2018 census data for the adult Swiss population [41]. We used direct standardization with age bands of 18–59, 60–69, 70–79, and ≥80 years. As TAVR is currently recommended by the European Society of Cardiology guidelines in patients aged ≥75 years [4], we also determined age-/sex-standardized TAVR and SAVR rates in patients aged <75 years vs. ≥75 years. To examine the variation in procedure rates across HSAs, we determined the extremal quotient (EQ), which is the highest divided by the lowest procedure rate. While the EQ is an intuitive measure of variation, it is prone to distortion by extreme values [42]. We also calculated the coefficient of variation (CV), i.e., the ratio of the standard deviation of the procedures rates to the mean rate, and the systematic component of variation (SCV) [42,43]. The SCV represents the non-random part of the variation while reducing the effect of extreme values [42–44]. An SCV of >5.4–10 is indicative of a high variation and an SCV of >10 of a very high variation [42,44].

### Determinants of variation

Differences in illness incidences and socioeconomic factors are legitimate causes of variation [42]. We therefore explored four regional domains that could influence procedure rates: demographics (age, sex), regional socioeconomic status (language region, median population density of persons aged ≥18 years, SSEP, percentage of discharges with [semi]private health insurance, and Swiss citizenship), population burden of disease, and supply factors (density of cardiologists/cardiovascular surgeons). Based on the language spoken by the majority of inhabitants, each HSA was classified as either Swiss German or French/Italian language region [45]. We used population density as a proxy for the level of urbanization of the area a resident lived in. As a measure for the population burden of disease, we calculated age-standardized incidence rates of hip fractures (ICD 10 codes S720-22), colon (ICD 10 codes C18/19 and CHOP codes 446 or 457–58) or lung cancer (ICD 10 codes C34 and CHOP codes 323-26/329) treated surgically, acute myocardial infarctions (ICD 10 codes I21), or strokes (ICD 10 codes I63/64) for each HSA, as differences in these disease rates are likely to reflect true regional differences in disease burden rather than differences in coding intensity or supply factors [46,47]. The density of cardiologists/cardiovascular surgeons was used as a supply measure.

### Statistical analyses

To explore determinants of procedure rates in Switzerland, we used progressively adjusted multilevel Poisson regression with a log link to model the procedure rates in each HSA. Model 1 included only the calendar year of the procedure. Model 2 was additionally adjusted for demographics (age and sex). Age was divided into the following bands: 18 to 54, 55 to 64, 65 to 74, 75 to 84, and 85+ years. Model 3 was further adjusted for HSA-level language region and insurance status. As population density, SSEP, and citizenship had a variance inflation factor of >5 indicating a high correlation with other variables, they were not included in the final model to avoid variance inflation and multi-collinearity of predictors [48]. Model 4 was further adjusted for HSA-level population burden of disease. Model 5 was additionally adjusted for the density of cardiologists and cardiovascular surgeons. HSA was included as a random intercept in all models. All covariates were selected *a priori*. We depicted the variation in HSA rates as average predicted procedure rates per 100,000 persons per HSA derived from the full multilevel regression models. Where rates are shown in maps, rate categories were chosen to be approximately equal in width.

We expressed the impact of determinants on procedure rates as incidence rate ratios (IRRs), defined as the procedure rate in the defined category (e.g., French/Italian language region) relative to the estimated procedure rate in the reference category (e.g., Swiss German language region). We also determined the percentage reduction in procedure variation across the eight HSAs by examining the variance of the random intercept relative to model 1. We considered the residual, unexplained variation of the fully adjusted model as a proxy for unwarranted variation that cannot be attributed to potential patient need [30,32,33]. We further assessed remaining variation in procedure rates across HSAs after full adjustment (model 5) using funnel plots. We plotted procedure rates against population size for each HSA. The mean procedure rates and the control limits of 2 and 3 standard deviations above and below the mean (95% and 99.8% confidence intervals), respectively, were calculated for all possible values for population size and used to create the funnel plot based on exact Poisson confidence intervals [49]. Statistical modeling was performed using Stata version 16.1 (StataCorp, College Station, TX). HSAs were delineated and maps drawn using the R statistical software, version 3.4.2. [50].

## Results

### Characteristics of procedure-specific HSAs and the study population

Five HSAs were located in Swiss German and three in the French/Italian-speaking part of Switzerland. The median population size aged ≥18 years was 525,164 persons (interquartile range [IQR] 409,016–1,149,208) per HSA, with a median population density of 165 persons/km^2^ (IQR 132–415). The mean proportion of residents with a (semi)private insurance was 25% (standard deviation 21–28%) and the median density of cardiologists/cardiovascular surgeons was 9 (IQR 7.1–10.0) per 100,000 persons.

**Fig 1:**
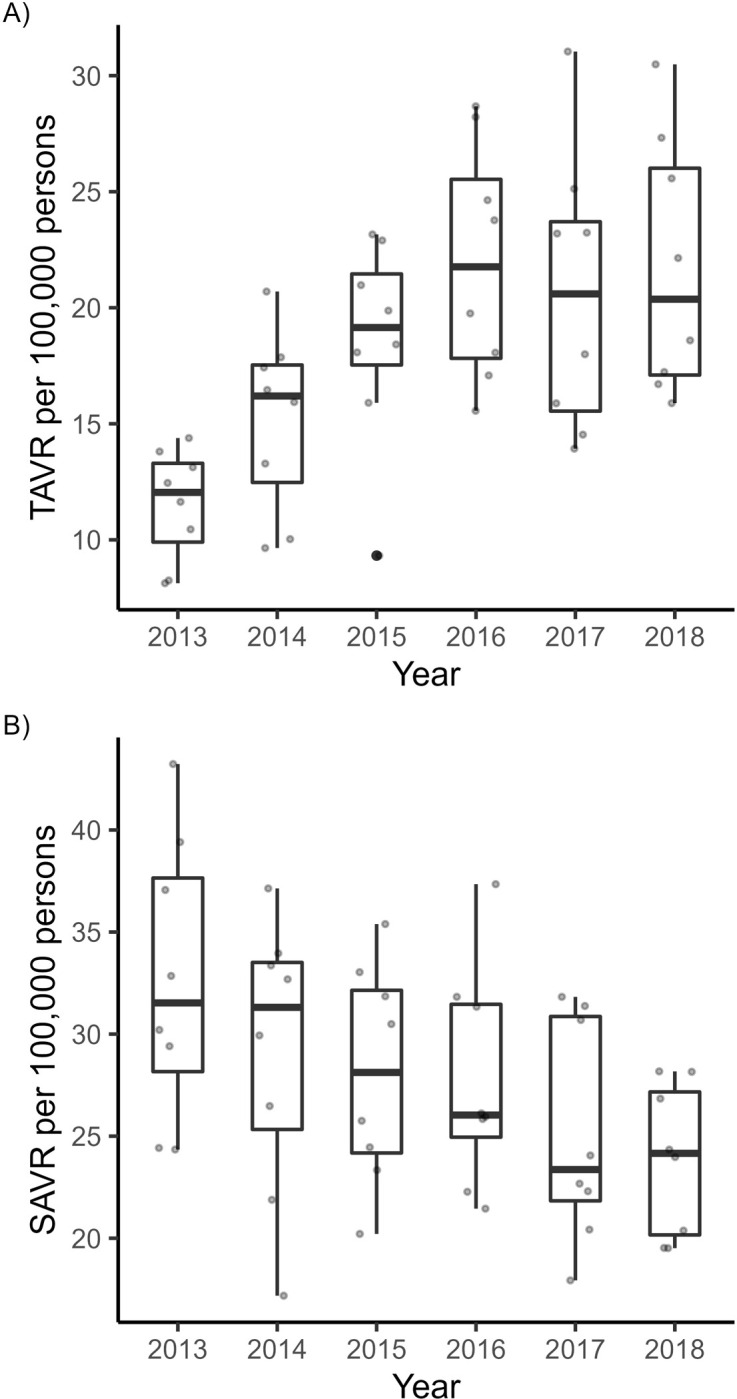
Annual age-/sex-standardized TAVR and SAVR rates by HSA from 2013 to 2018. Boxplot depicting age-/sex-standardized procedures rates, overlaid with the individual datapoints.

Overall, 8074 TAVR and 11,825 SAVR procedures were performed between 2013 and 2018. The national age-/sex-standardized aortic valve replacement rate (TAVR or SAVR) increased slightly from 44 to 46 per 100’000 persons between 2013 and 2018 with TAVR rates increasing from 12 to 22 per 100,000 persons (**Fig 1, Panel A**) and SAVR rates decreasing from 33 to 24 per 100,000 persons during the same period (**Fig 1, Panel B**). TAVR were mainly performed in persons aged ≥75 years (87%) whereas SAVR were mainly done in persons aged <75 years (71%). Patient characteristics by procedure type are shown in **Table 1**.

**Table 1:** Characteristics of patients undergoing TAVR or SAVR from 2013 to 2018 (N = 19,899).

	TAVR (N = 8074)	SAVR (N = 11,825)
	n (%)	
Age, years		
18–54	55 (1)	1650 (14)
55–64	163 (2)	2261 (19)
65–74	857 (11)	4498 (38)
75–84	4373 (54)	3212 (27)
≥85	2626 (33)	204 (2)
Sex		
Male	4113 (51)	8462 (72)
Insurance class		
General	5534 (69)	8407 (71)
(Semi)private	2540 (31)	3416 (29)
Unknown	2 (0)	0 (0)
French/Italian language region	1413 (18)	2888 (24)
Swiss citizenship	7395 (92)	10428 (88)

Abbreviations: TAVR = transcatheter aortic valve replacement; SAVR = surgical aortic valve replacement.

### Variation in procedure rates across HSAs

The mean overall age-/sex-standardized TAVR rate from 2013 to 2018 was 18 (range 13–23) per 100,000 persons across the eight HSAs, with a rate of 3 (range 1–4) per 100,000 persons in persons aged <75 years and 160 (range 109–206) per 100,000 persons in those aged ≥75 years. Detailed unadjusted, age-/sex-standardized, and fully adjusted TAVR rates by HSA are shown in the **S1 Table.**

**Fig 2:**
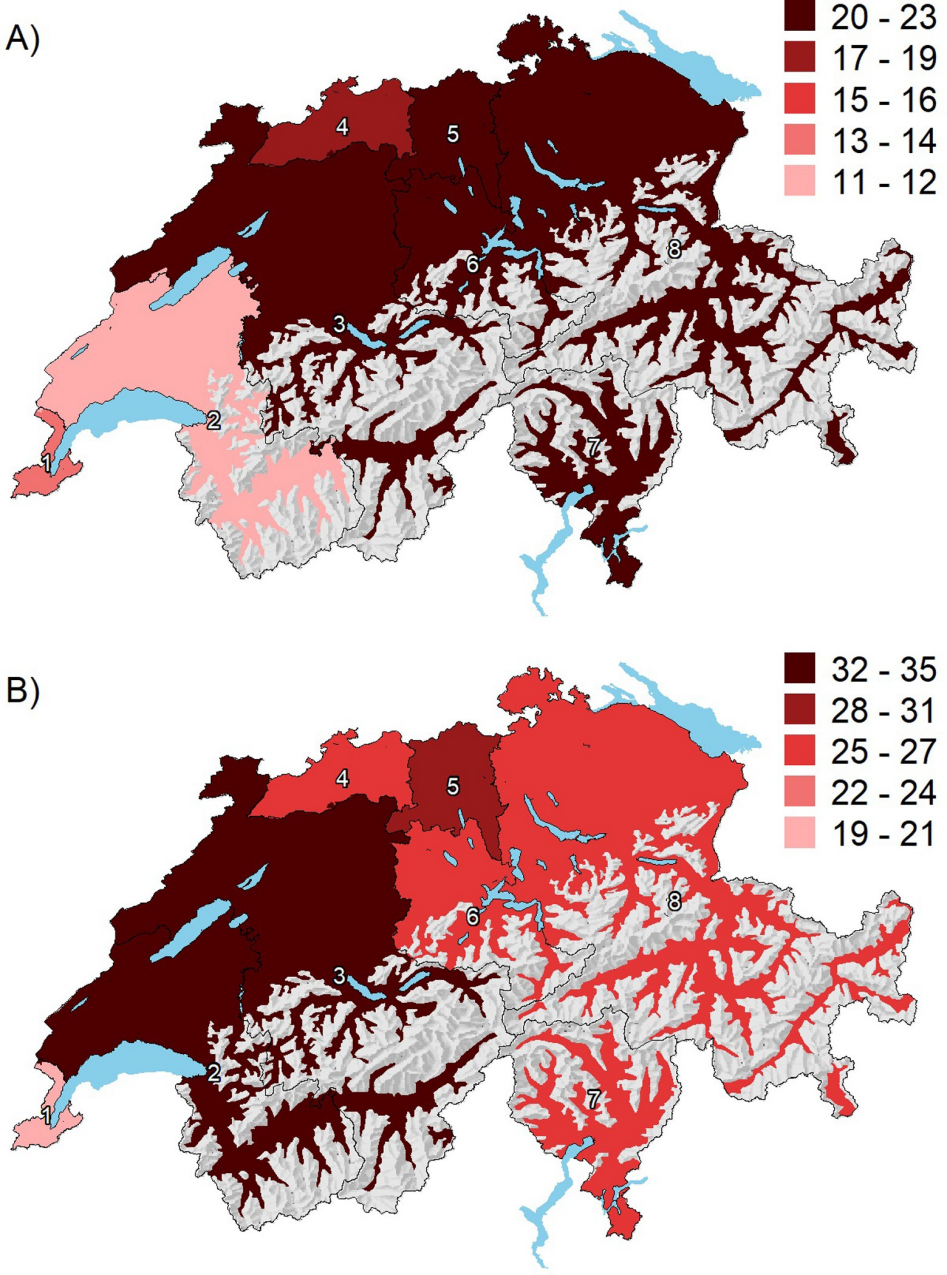
Average predicted TAVR (A) and SAVR (B) rates across 8 Swiss HSAs. Abbreviation: HSA = hospital service area. Average predicted procedures rates for each HSA are shown as red-scale categories per 100,000 per-sons. Adjusted for procedure year and population age, sex, language, insurance, burden of disease, and density of cardiologists/cardiovascular surgeons. Shaded relief map reprinted from the Federal Office of Topography swisstopo, Switzerland https://shop.swisstopo.admin.ch/en/products/maps/overview/relief and shape files derived from post-code-level shape file used to create map of Switzerland, e.g., https://www.geocat.admin.ch/) under a CC BY license, with permission from Alexandra Frank, original copyright 2006.

The variation in overall procedure rates (TAVR or SAVR) across HSAs was low between 2013 and 2018 (EQ 1.5, CV 0.1, SCV 1.6) and remained low over time (**Table 2**). The EQ for TAVR was 1.9 (age <75 years: 2.9; age ≥75 years: 1.9), the CV 0.2 (<75 years: 0.4; ≥75 years: 0.2), and the SCV 3.9 (<75 years: 9.5; ≥75 years: 3.6) (**Table 2**). The regional variation in TAVR increased between 2013 and 2018 (SCV from 2.1 to 4.5). After full adjustment for procedure year, age, sex, language region, insurance status, burden of disease, and density of cardiologists/cardiovascular surgeons, the predicted TAVR rates varied between 12 and 22 per 100,000 persons across the eight HSAs (**Fig 2, Panel A**). Average predicted TAVR rates in patients aged <75 and ≥75 years after full adjustment are shown in **S1 Fig (Panel A)**.

**Table 2:** Measures of variation in age-/sex-standardized TAVR and SAVR rates across 8 Swiss HSAs from 2013 to 2018.

	Overall	2013	2014	2015	2016	2017	2018
**All procedures**							
**EQ**	1.5	1.6	1.9	1.4	1.5	1.9	1.6
**CV**	0.1	0.2	0.2	0.1	0.1	0.2	0.1
**SCV**	1.6	2.0	3.5	1.0	1.1	3.2	1.3
**TAVR**							
EQ	1.9	1.8	2.2	2.5	1.8	2.2	1.9
CV	0.2	0.2	0.3	0.2	0.2	0.3	0.3
SCV	3.9	2.1	4.4	3.9	3.6	5.9	4.5
**SAVR**							
EQ	1.6	1.8	2.2	1.8	1.7	1.8	1.4
CV	0.2	0.2	0.2	0.2	0.2	0.2	0.2
SCV	2.2	3.2	4.0	2.5	2.4	3.3	1.5

Abbreviations: TAVR = transcatheter aortic valve replacement; SAVR = surgical aortic valve replacement; HSA = hospital service area; EQ = extremal quotient; CV = coefficient of variation; SCV = systematic component of variation.

The mean age-/sex-standardized rate for SAVR was 28 (range 22–34) per 100,000 persons across the eight HSAs, with a rate of 22 (range 17–27) per 100,000 persons in persons aged <75 years and 79 (range 63–123) per 100,000 persons in those aged ≥75 years. Detailed unadjusted, age-/sex-standardized, and fully adjusted SAVR rates by HSA are shown in the **S1 Table.** The EQ was 1.6 (age <75 years: 1.6; age ≥75 years: 1.9), the CV 0.2 (<75 years: 0.2; ≥75 years: 0.2), and the SCV 2.2 (age <75 years: 2.3; age ≥75 years: 4.6) (**Table 2**). From 2013 to 2018, the regional variation in SAVR decreased over time (SCV from 3.2 to 1.5). After full adjustment, the predicted SAVR rates varied between 20 and 35 per 100,000 persons across HSAs (**Fig 2, Panel B**). Average predicted SAVR rates in patients aged <75 and ≥75 years after full adjustment are shown in **S1 Fig** (**Panel B)**.

### Determinants of variation in procedure rates

Whereas TAVR procedure rates increased annually by 10%, SAVR rates decreased by 5% per year between 2013 and 2018 (**Table 3**). Compared to persons aged 55–64 years, persons aged ≥85 years had an 88-fold higher TAVR and a 46% lower SAVR rate. Women were less likely to receive aortic valve replacement than men (TAVR -34%; SAVR -65%). Residence in a French/Italian language region was associated with a lower likelihood of TAVR (-21%) than in Swiss German regions. A 10% higher regional proportion of (semi)private insurance coverage was associated with a 40% increase in TAVR rates and a 20% decrease in SAVR rates. An increase of 1 comorbidity per 1000 persons was associated with a 4-fold increase in TAVR. A higher density of cardiologists/cardiovascular surgeons was associated with lower TAVR rates (-5%). During stepwise adjustment, most variance was explained by language region and insurance status (TAVR 59.6%, SAVR 60.6% of the variance, model 3, **Table 4**) and burden of disease in SAVR (11.5%, model 4). Overall, 10.6% of the variance in TAVR and 18.4% of the variance in SAVR remained unexplained. The unexplained variance in TAVR was larger in patients aged <75 vs. ≥75 years (33.3% vs. 9.7%) but did not differ much in SAVR (12.9% vs. 14.5%).

**Table 3:** Regional determinants of variation in incidence rates of TAVR and SAVR across 8 Swiss HSAs.

	TAVR	SAVR
	IRR* (95% CI)	
Procedure year (per year)	**1.10 (1.08–1.12)**	**0.95 (0.93–0.96)**
Age 18–54	**0.08 (0.06–0.11)**	**0.18 (0.17–0.19)**
55–64	Reference	Reference
65–74	**6.95 (5.87–8.21)**	**2.64 (2.51–2.77)**
75–84	**58.63 (50.15–68.56)**	**3.23 (3.06–3.41)**
≥85	**87.85 (74.98–102.93)**	**0.54 (0.47–0.63)**
Sex Men	Reference	Reference
Women	**0.66 (0.63–0.69)**	**0.35 (0.34–0.37)**
Language region Swiss German	Reference	Reference
French/Italian	**0.79 (0.68–0.91)**	1.01 (0.88–1.15)
(Semi)private insurance (per 10% change)†	**1.40 (1.20–1.63)**	**0.80 (0.69–0.91)**
Burden of disease (per 1 per 1000)§	**4.04 (1.98–8.24)**	0.57 (0.31–1.03)
Density of cardiologists/cardiovascular surgeons (per 1 per 100,000)#	**0.95 (0.92–0.98)**	0.99 (0.96–1.02)

Abbreviations: TAVR = transcatheter aortic valve replacement; SAVR = surgical aortic valve replacement; HSA = hospital service area; IRR = incidence rate ratio; CI = confidence interval. Results in **bold** indicate a statistically significant effect.

*Procedure rate in the defined category relative to the procedure rate in the reference category. For instance, an IRR of 0.66 indicates a 34% lower TAVR rate in women than in men.

†The IRR is the increase (decrease) in procedure rates when the percentage of persons with (semi)private insurance changes by 10%.

§Burden of disease represents the sum of age-standardized incidence rates of hip fracture, colon or lung cancer treated surgically, acute myocardial infarction, and stroke. The IRR is the increase (decrease) in procedure rates when the regional burden of disease increases by 1 comorbidity per 1000 persons.

#The IRR is the increase (decrease) in procedure rates when the number of cardiologists/cardiovascular surgeons increases by 1 per 100,000 persons.

**Table 4:** Remaining variance in procedure rates after incremental adjustment*.

	Model 2†	Model 3‡	Model 4§	Model 5#
	**% of remaining variance**			
TAVR	87.7	28.1	32.8	10.6
<75 years	92.4	65.1	69.7	33.3
≥75 years	100.7	25.4	28.4	9.7
SAVR	91.4	30.8	19.3	18.4
<75 years	78.0	20.7	13.4	12.9
≥75 years	102.6	24.4	15.2	14.5

Abbreviations: TAVR = transcatheter aortic valve replacement; SAVR = surgical aortic valve replacement.

*Reference: model 1, adjustment for procedure year only.

†Adjusted for age and sex.

‡Additionally adjusted for language and insurance.

§Additionally adjusted for burden of disease.

#Additionally adjusted for density of cardiologists/cardiovascular surgeons.

When plotting procedure rates against population size, HSA 3 had a high TAVR rate (above the outer control limits of 2 standard deviations) (**S2 Fig, Panel A**) and a very high SAVR rate (above the outer control limits of 3 standard deviations) (**S2 Fig, Panel B**). HSA 8 had a high TAVR rate and a low SAVR rate. In contrast, HSA 2 had an unusually low TAVR rate and a high SAVR rate, and HSA 1 had low TAVR rate and an unusually low SAVR rate. All HSAs with high TAVR rates were located in the Swiss German speaking part of Switzerland and HSAs with low to very low TAVR rates were located in the French/Italian speaking part (**S2 Table**). The regional proportion of (semi)private insurance was not associated with unusually high or low TAVR rates (high proportion in HSA 8 and 1, with HSA 8 having a high TAVR rate and HSA 1 a low TAVR rate). In HSA 1, a lower burden of disease was observed compared to the other HSAs, which may explain the lower TAVR and the unusually low SAVR rate despite a higher density of cardiologists/cardiovascular surgeons.

## Discussion

Our study shows an annual 10% increase in TAVR and a 5% decrease in SAVR from 2013 to 2018, with an increasing regional variation in TAVR procedure rates over time. Age, sex, Swiss German language region, and a higher regional disease burden were associated with higher TAVR rates. A higher regional proportion of (semi)private insurance was associated with higher TAVR and lower SAVR rates. The most important predictors of SAVR performance were age and male sex. Regional differences in socioeconomic factors (language, insurance status) explained most of the variance in aortic valve replacement.

The annual increase in TAVR rates between 2013 and 2018 were paralleled by a decrease in SAVR rates, most probably due to a progressive shift towards TAVR use in patients with an intermediate to high surgical risk, and even in those with a low surgical risk. Indeed, a trend analysis of Swiss registry data showed a significant decrease in the baseline surgical risk of patients undergoing TAVR from 2011 to 2015 [51]. Equal clinical outcomes of TAVR as compared to SAVR independent of the surgical risk and higher patient satisfaction (i.e., through less invasive procedure, faster recovery) may increase future TAVR use at the expense of SAVR [27,52]. Consistent with recommendations that TAVR should be mainly performed in older, multimorbid patients [4,5,53], we found a strong association between older age, disease burden, and TAVR performance.

Although bicuspid aortic valves are 3–4 times more common in men [54], the incidence and progression of degenerative AS appears to be comparable in women and men [55–57]. In our study, women had a 34% lower likelihood to undergo TAVR and 65% lower likelihood to undergo SAVR than men. While women were also less likely to undergo aortic valve replacement in Australia between 2004 and 2019 [24], women had higher TAVR rates compared to men in the U.S. [26]. Overall, sex-related differences in the invasive treatment of AS are poorly understood. A possible explanation for the lower SAVR performance rates in women may be their potentially higher mortality risk when undergoing SAVR [58], as women undergoing this procedure have a higher risk profile [59]. A meta-analysis of randomized trials showed a 1-year mortality reduction of 31% in women undergoing TAVR compared to SAVR, and no difference in men [60,61]. The reasons for the lower TAVR/SAVR rates in women in Switzerland warrant further studies.

French/Italian speaking areas had a 21% lower TAVR rate than Swiss German regions, potentially due to a more conservative physician practice style in these areas. We previously found lower rates of several preference-sensitive interventions in French/Italian speaking Swiss regions compared to Swiss German speaking areas, including vertebro-/kyphoplasty, hysterectomy, and joint arthroplasty [30,32,33].

We found a 40% higher TAVR rate in regions with a higher prevalence of (semi)private insurance, raising the question whether additional insurance (which results in higher hospital and physician reimbursement) may influence the decision to perform TAVR. In 2014, the average reimbursement was CHF 72,000 (device cost CHF 32,000) for TAVR and CHF 43,000 for SAVR (device cost CHF 3000) in Switzerland [62]. While the higher reimbursement for TAVR may be offset by the substantially higher device costs, TAVR may be more cost-effective than SAVR in low-intermediate risk patients due to a reduction in hospital days and rehabilitation/skilled nursing days [63]. Moreover, TAVR device costs decreased between 10–15% in Switzerland from 2012 to 2014 [62]. Further reductions in device costs would make TAVR even more cost-effective. A socioeconomic analysis across European countries showed a linear correlation between the number of TAVR implants and healthcare spending [25], indicating that TAVR may be less affordable in countries with a lower spending.

In contrast to a previous Swiss study that showed a general association between overall physician density and health expenditures due to a possible supply-induced demand [64], we found an inverse association between the regional density of cardiologists/cardiovascular surgeons and increased TAVR procedure rates. First, assuming similar patient preferences for TAVR across Swiss regions, it is likely that the remaining variance in these procedure rates is not primarily related to differing specialist physician numbers but due to differences in local physician practice patterns, including individual physicians’ attitudes and the speed/extent of adoption of new technologies by highly specialized heart teams in tertiary-care facilities [65]. Second, because we had no information about the number of cardiologists/cardiovascular surgeons who actually performed TAVR/SAVR procedures, it is possible that the number of specialty title holders only imperfectly reflects the number of interventionalists.

Interestingly, the unexplained variation in TAVR was substantially higher in patients aged <75 (33.3%) after full adjustment. Conversely, unexplained practice variation in TAVR in patients aged ≥75 years and in SAVR was low and unexplained variation in SAVR was almost identical in younger vs. older patients. A potential explanation is that guidelines explicitly recommend TAVR as the preferred option in older patients, thus reducing practice variation in this age group [66].

Our study had several limitations. First, we analyzed anonymized administrative data and had no clinical data on patient symptoms, severity of AS, or individual comorbidities. Thus, we could not verify the appropriateness of procedure performance. Second, we had no information about other potential drivers of regional variation in TAVR/SAVR procedures, including differences in patient preferences, physician attitudes, and technical expertise. The impact of these factors on aortic valve replacement procedures should be further explored. Third, although we adjusted for burden of disease on a population level, we had no information on the prevalence of AS in the population. Thus, we cannot entirely exclude the possibility that geographical variations in procedure rates are at least partially due to regional variation in the prevalence of AS across Swiss HSAs. However, the regional variation of cardiovascular risk factors is low across Switzerland [67,68], and the prevalence of AS is very unlikely to differ across geographically close HSAs between which we observed substantial procedure variation (e.g., HSA 1 and 2). Fourth, we could not stratify patients with AS according to their surgical risk and thus we were not able to examine procedure variation in these subgroups. Fifth, as our data was limited to calendar years 2013 to 2018 at the time of analysis, we could not examine more recent procedure trends. Finally, adjustment for ecological variables on a population level (i.e., age, language, etc.) carries a risk of ecological fallacy by drawing conclusions about individuals based on population parameters [69].

In conclusion, we found a low to moderate overall and annual regional variation in TAVR and SAVR procedure rates, with an increasing use of TAVR at the expense of more invasive SAVR across Swiss HSAs between 2013 and 2018. Several regional factors, particularly demographics, language, insurance, and disease burden were associated with procedure rates. After full adjustment, 10.6% of the variation in TAVR and 18.4% of the variation in SAVR remained unexplained and may represent unwarranted variation due to differing physicians’ styles and/or affinity towards new technologies.

## Supporting information

S1 Fig Average predicted TAVR procedure rates across 8 Swiss HSAs. Panel A: Patients aged <75 years, Panel B: Patients aged ≥75 years. Abbreviations: uninhab. = uninhabited area; HSA = hospital service area. Average predicted TAVR rates for each HSA are shown as red-scale categories per 100,000 persons. Adjusted for procedure year, population age, sex, language, insurance, burden of disease, and the density of cardiologists/cardiovascular surgeons. Shaded relief map reprinted from the Federal Office of Topography swisstopo, Switzerland https://shop.swisstopo.admin.ch/en/products/maps/overview/relief and shape files derived from postcode-level shape file used to create map of Switzerland, e.g., https://www.geocat.admin.ch/) under a CC BY license, with permission from Alexandra Frank, original copyright 2006.
(TIF)

S2 Fig Funnel plot analysis of the variation in fully adjusted TAVR and SAVR rates across 8 HSAs. Panel A: TAVR rates, Panel B: SAVR rates. The straight line indicates the mean procedure rate; the inner and outer control limits represent 2 and 3 standard deviations above/below the mean, respectively (i.e., 95% and 99.8% of data, respectively). Thus, of eight HSAs, <0.1 could be expected to lie beyond the outer control limit by chance; 0.4 HSAs could be expected to lie above or below the inner control limit by chance. HSAs with procedure rates that fall out of the outer control limits are tagged by their given HSA-number indicating potential over- or under-treatment in the specific HSAs respectively.
(TIF)

S1 Table TAVR and SAVR rates by HAS. Abbreviations: TAVR = transcatheter aortic valve replacement; SAVR = surgical aortic valve replacement; HSA = hospital service area; CI = confidence interval. *Adjusted for procedure year and population age, sex, language, insurance, burden of disease, and density of cardiologists/cardiovascular surgeons.
(DOCX)

S2 Table Characteristics of HSAs with TAVR and SAVR rates outside the 95% confidence interval funnel plot. Abbreviations: HSA = hospital service area; TAVR = transcatheter aortic valve replacement; SAVR = surgical aortic valve replacement; CI = confidence interval. High/low rates indicate rates above/below the 95% confidence interval; unusual high/low rates indicate rates above/below the 99.8% confidence interval. *Adjusted for procedure year and population age, sex, language, insurance, burden of disease, and density of cardiologists/cardiovascular surgeons. §Language region within Switzerland, i.e., Swiss German or French/Italian speaking region. #Burden of disease represents the sum of age-standardized regional incidence rates for the following comorbidities: hip fracture, colon or lung cancer treated surgically, acute myocardial infarction, and stroke.
(DOCX)
