# Supplementary figures and images for: Regional variation and temporal trends in transcatheter and surgical aortic valve replacement in Switzerland: A population-based small area analysis
Source: PLoS One. 2024 Jan 8;19(1):e0296055. doi: 10.1371/journal.pone.0296055 (PMC10773935; doi:10.1371/journal.pone.0296055)

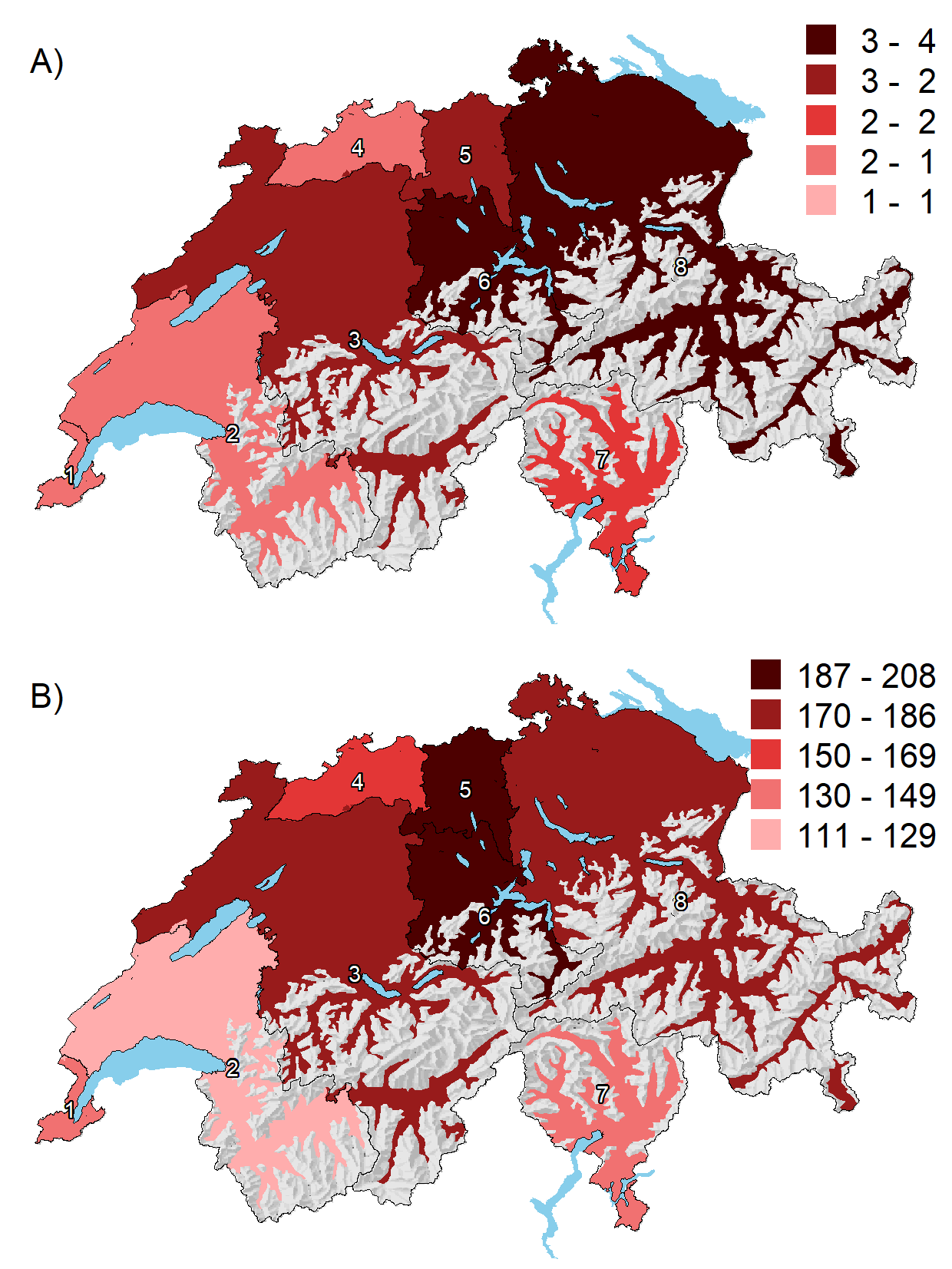

Supplement: S1 Fig — Abbreviations: uninhab. = uninhabited area; HSA = hospital service area. Average predicted TAVR rates for each HSA are shown as red-scale categories per 100,000 persons. Adjusted for procedure year, population age, sex, language, insurance, burden of disease, and the density of cardiologists/cardiovascular surgeons. Shaded relief map reprinted from the Federal Office of Topography swisstopo, Switzerland https://shop.swisstopo.admin.ch/en/products/maps/overview/relief and shape files derived from postcode-level shape file used to create map of Switzerland, e.g., https://www.geocat.admin.ch/) under a CC BY license, with permission from Alexandra Frank, original copyright 2006. (TIF) [file pone.0296055.s001.tif]

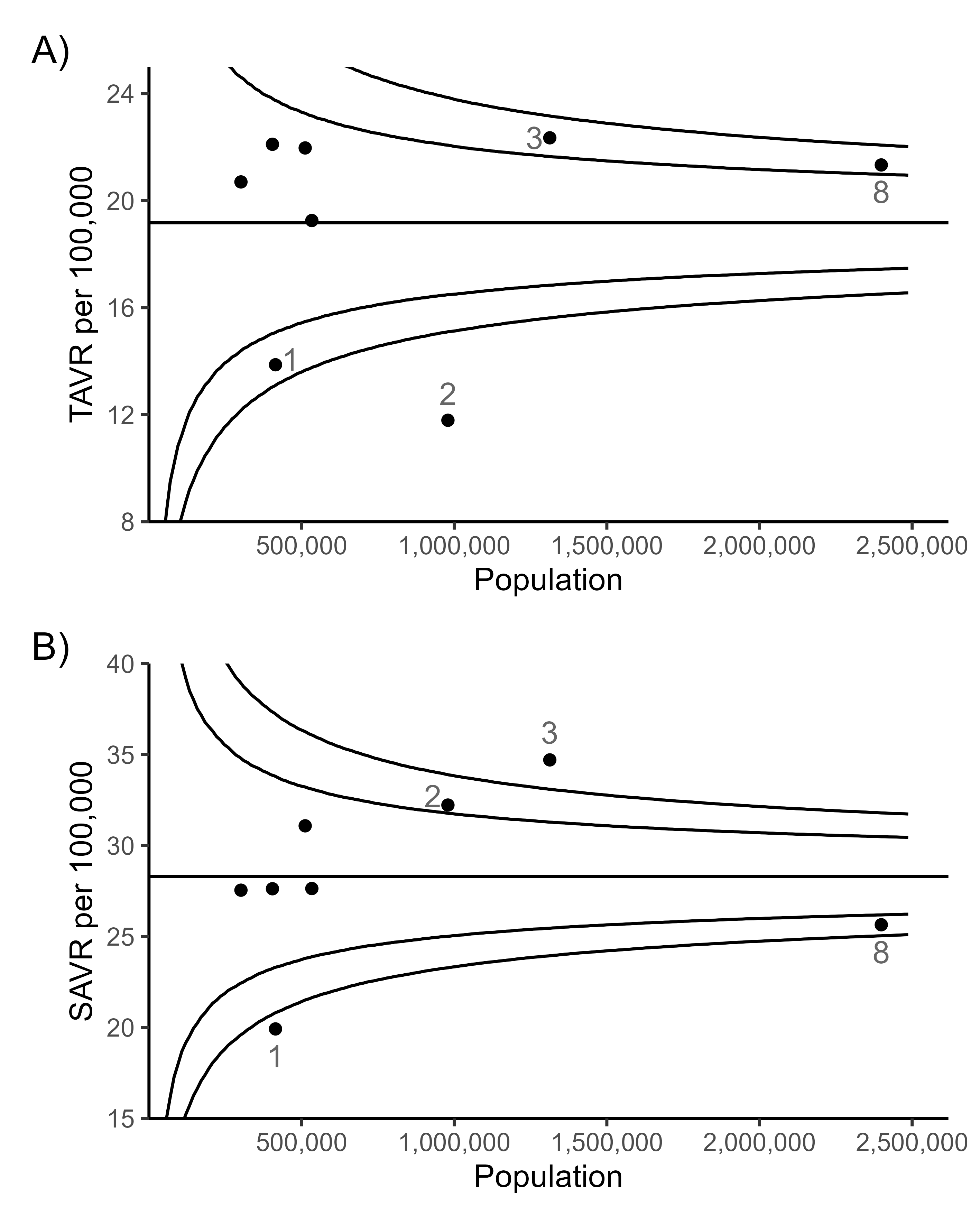

Supplement: S2 Fig — The straight line indicates the mean procedure rate; the inner and outer control limits represent 2 and 3 standard deviations above/below the mean, respectively (i.e., 95% and 99.8% of data, respectively). Thus, of eight HSAs, <0.1 could be expected to lie beyond the outer control limit by chance; 0.4 HSAs could be expected to lie above or below the inner control limit by chance. HSAs with procedure rates that fall out of the outer control limits are tagged by their given HSA-number indicating potential over- or under-treatment in the specific HSAs respectively. (TIF) [file pone.0296055.s002.tif]
