# Supplementary material for: Regional variation and temporal trends in transcatheter and surgical aortic valve replacement in Switzerland: A population-based small area analysis
Source: PLoS One. 2024 Jan 8;19(1):e0296055. doi: 10.1371/journal.pone.0296055 (PMC10773935; doi:10.1371/journal.pone.0296055)
Supplement: S1 Table — Abbreviations: TAVR = transcatheter aortic valve replacement; SAVR = surgical aortic valve replacement; HSA = hospital service area; CI = confidence interval. *Adjusted for procedure year and population age, sex, language, insurance, burden of disease, and density of cardiologists/cardiovascular surgeons. (DOCX) [file pone.0296055.s003.docx]

**S1 Table. TAVR and SAVR rates by HSA**

| **HSA** | **TAVR rates per 100,000 persons**  **(95% CI)** | | | **SAVR rates per 100,000 persons**  **(95% CI)** | | |
| --- | --- | --- | --- | --- | --- | --- |
|  | **Crude** | **Age-/sex-standardized** | **Fully**  **adjusted*** | **Crude** | **Age-/sex-standardized** | **Fully**  **adjusted*** |
| 1 | 14.0 | 14.4 | 13.9 (11.4 – 16.9) | 20.2 | 21.7 | 19.9 (16.8 – 23.7) |
| 2 | 11.7 | 12.5 | 11.8 (10.0 – 13.8) | 32.5 | 34.3 | 32.2 (28.1 – 36.9) |
| 3 | 22.7 | 20.4 | 22.3 (19.6 – 25.5) | 34.9 | 32.6 | 34.7 (30.9 – 39.0) |
| 4 | 18.7 | 15.8 | 19.3 (16.7 – 22.2) | 27.3 | 25.5 | 27.6 (24.4 – 31.3) |
| 5 | 21.6 | 23.3 | 22.0 (19.1 – 25.3) | 31.7 | 31.3 | 31.1 (27.5 – 35.1) |
| 6 | 22.5 | 22.7 | 22.1 (18.8 – 26.0) | 27.0 | 26.8 | 27.6 (23.9 – 31.9) |
| 7 | 20.7 | 16.5 | 20.7 (17.5 – 24.4) | 26.4 | 23.3 | 27.5 (23.8 – 31.9) |
| 8 | 21.3 | 21.7 | 21.3 (19.3 – 23.6) | 25.6 | 25.7 | 25.6 (23.4 – 28.1) |

Abbreviations: TAVR= transcatheter aortic valve replacement; SAVR= surgical aortic valve replacement; HSA= hospital service area; CI= confidence interval.

*Adjusted for procedure year and population age, sex, language, insurance, burden of disease, and
