# Supplementary material for: Regional variation and temporal trends in transcatheter and surgical aortic valve replacement in Switzerland: A population-based small area analysis
Source: PLoS One. 2024 Jan 8;19(1):e0296055. doi: 10.1371/journal.pone.0296055 (PMC10773935; doi:10.1371/journal.pone.0296055)
Supplement: S2 Table — Abbreviations: HSA = hospital service area; TAVR = transcatheter aortic valve replacement; SAVR = surgical aortic valve replacement; CI = confidence interval. High/low rates indicate rates above/below the 95% confidence interval; unusual high/low rates indicate rates above/below the 99.8% confidence interval. *Adjusted for procedure year and population age, sex, language, insurance, burden of disease, and density of cardiologists/cardiovascular surgeons. §Language region within Switzerland, i.e., Swiss German or French/Italian speaking region. #Burden of disease represents the sum of age-standardized regional incidence rates for the following comorbidities: hip fracture, colon or lung cancer treated surgically, acute myocardial infarction, and stroke. (DOCX) [file pone.0296055.s004.docx]

**S2 Table. Characteristics of HSAs with TAVR and SAVR rates outside the 95% confidence interval funnel plot**

| **HSA** | **Fully adjusted***  **TAVR rate per 100,000 persons (95% CI)** | **Fully adjusted* SAVR rate per 100,000 persons (95% CI)** | **Language**  **region§** | **(Semi)private insurance (%)** | **Burden of disease**  **(per 1 per 1000 persons)#** | **Density of cardiologists/ cardiovascular surgeons**  **(per 1 per 100,000 persons)** |
| --- | --- | --- | --- | --- | --- | --- |
| High TAVR rate and unusual high SAVR rate | | | | | | |
| 3 | 22.3 (19.6 - 25.5) | 34.7 (30.9 – 39.0) | Swiss German | 18.91 | 1.10 | 8.94 |
| High TAVR rate and low SAVR rate | | | | | | |
| 8 | 21.3 (19.3 – 23.6) | 25.6 (23.4 - 28.1) | Swiss German | 27.68 | 1.03 | 8.80 |
| Unusually low TAVR rate and high SAVR rate | | | | | | |
| 2 | 11.8 (10.0 – 13.8) | 32.2 (28.1 - 36.9) | French/Italian | 19.05 | 1.03 | 9.00 |
| Low TAVR rate and unusual low SAVR rate | | | | | | |
| 1 | 13.9 (11.4 - 16.9) | 19.9 (16.8 – 23.7) | French/Italian | 39.75 | 0.85 | 15.35 |

Abbreviations: HSA= hospital service area; TAVR= transcatheter aortic valve replacement; SAVR= surgical aortic valve replacement; CI= confidence interval.

High/low rates indicate rates above/below the 95% confidence interval; unusual high/low rates indicate rates above/below the 99.8% confidence interval.

*Adjusted for procedure year and population age, sex, language, insurance, burden of disease, and density of cardiologists/cardiovascular surgeons.

§Language region within Switzerland, i.e., Swiss German or French/Italian speaking region.

#Burden of disease represents the sum of age-standardized regional incidence rates for the following comorbidities: hip fracture, colon or lung cancer treated surgically, acute myocardial infarction, and stroke.
